# Two Forms of Electrical Transmission Between Neurons

**DOI:** 10.3389/fnmol.2018.00427

**Published:** 2018-11-21

**Authors:** Donald S. Faber, Alberto E. Pereda

**Affiliations:** ^1^Dominick P. Purpura Department of Neuroscience, Albert Einstein College of Medicine, New York, NY, United States; ^2^Marine Biological Laboratory, Woods Hole, MA, United States

**Keywords:** synaptic communication, electrical synapse, gap junction, electric field, ephapsis

## Abstract

Electrical signaling is a cardinal feature of the nervous system and endows it with the capability of quickly reacting to changes in the environment. Although synaptic communication between nerve cells is perceived to be mainly chemically mediated, electrical synaptic interactions also occur. Two different strategies are responsible for electrical communication between neurons. One is the consequence of low resistance intercellular pathways, called “gap junctions”, for the spread of electrical currents between the interior of two cells. The second occurs in the absence of cell-to-cell contacts and is a consequence of the extracellular electrical fields generated by the electrical activity of neurons. Here, we place present notions about electrical transmission in a historical perspective and contrast the contributions of the two different forms of electrical communication to brain function.

## Introduction

It has been argued that the function of the nervous system is to support movement and that it evolved because of its usefulness to organisms in navigating their environment (Llinás, 2001). Early observations established that nerves were required for muscle contraction. However, the mechanism underlying this interaction was unknown. An old, predominant, idea embraced by Rene Descartes was that muscle contraction resulted from the action of “animal spirits” running through hollow nerves (Piccolino, 1998; Finger, 2005). This and other speculative ideas were later disproved, leading to the consideration of alternative mechanisms. One of them was electricity (Franklin, 1751). The use of electricity for therapeutic purposes was popular in the second part of the 18th century, and electricity was capable of eliciting muscle contraction. In addition, because of its high travel velocity, electricity was ideally suited to be the agent responsible for nerve action, as some hypothesized (Finger, 2005). Furthermore, experimental evidence showed that certain fish were capable of generating electricity. All this preceding work and speculations paved the way to the studies conducted by Galvani (1791) which demonstrated that nerves and muscles generate electricity (“bioelectricity”) and, therefore, that electricity was the mysterious fluid or “animal spirit” responsible for nerve conduction and muscle contraction (Piccolino, 1998; Finger, 2005). We know now that these electrical currents result from the movement of charged ions across the cellular membrane following their electrochemical gradient (Hodgkin and Huxley, 1952; Armstrong, 2007). Galvani’s seminal studies led to the foundation of electrophysiology and to the discovery that brain function and, hence, animal behavior, depends upon electrophysiological computations, the only operational mode fast enough to support the required time frame of decision making by neural circuits. In other words, as emphasized by Llinás, electricity makes us who we are (Sohn, 2003).

The discovery that the brain is constructed from networks of individual cells that generate electrical signals raised the question of how electrical currents “jump” from one cell to another. The most hotly debated question in Neuroscience during the 20th century was whether synaptic transmission, which is the currency of the brain, is mediated electrically or chemically. In fact, this might have been the major point of dispute in the biological sciences in that era, with advocates on both sides avidly defending their positions with data—based and theoretical models. Each side advanced its favored mechanism on the basis of its assumed advantages for the operation of neural networks in the central nervous system (CNS). Thus, a great deal of effort was devoted to determining whether there was a delay of 1–2 ms between a presynaptic action potential and the start of a postsynaptic response (chemical) or not (electrical), and to the corresponding functional consequences of these alternatives. In this review article, we briefly describe the critical elements of the debate between electrical and chemical modes of transmission, which seemed to tilt strongly in favor of the latter once it emerged that synaptic inhibition in the spinal cord was mediated by an ionic conductance change. This was particularly compelling in view of the difficulties in determining a satisfying mechanism for electrical inhibition. However, in recent years, electrical transmission has regained recognition and relevance. Rather than occurring via a single mechanism, electrical transmission operates in two ways: via pathways of low resistance between neurons (gap junctions) or as a consequence of extracellular electric fields generated by neuronal activity. Thus, we focus not only on the differences between these modes of operation, but also on the concept they share some operational characteristics. Far from providing an extensive review on the topic, we center here on a number of classic and recent examples that we believe illustrate these properties.

## The Search for the Mechanisms of Synaptic Transmission

**Figure 1:**
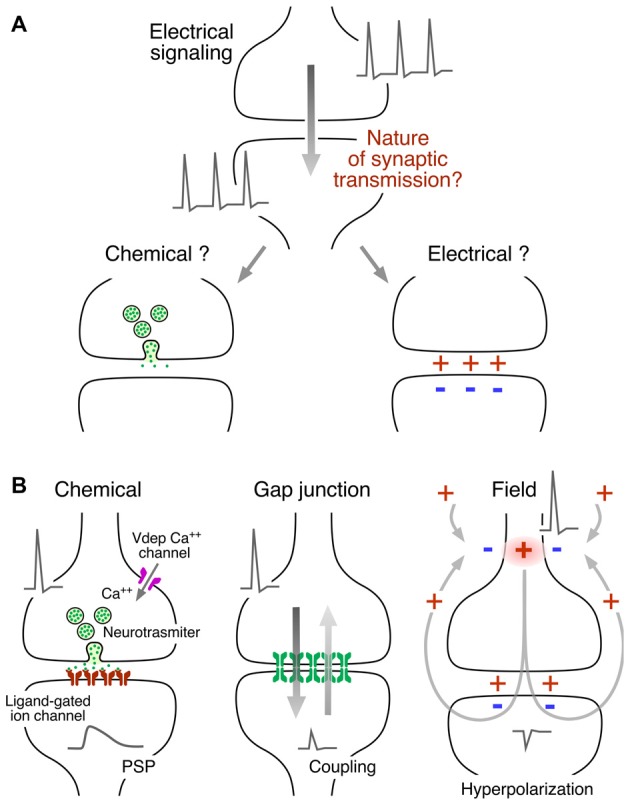
Mechanisms of synaptic communication between neurons. **(A)** Neurons operate electrically (action potentials at pre- and postsynaptic neurons) but the nature of the mechanism of neuronal intercommunication was a source of controversy. The interposition of a chemical messenger or the existence of electrical interactions were proposed to explain synaptic transmission. **(B)** Both, chemical and electrically-mediated mechanisms of communication were later found to co-exist in all nervous systems. Left: chemical transmission represents an electrically-regulated Ca^++^-dependent form of release. An action potential provides the depolarization required for the activation of voltage-dependent Ca^++^ channels, the source of the Ca^++^ influx in the presynaptic terminal. The released neurotransmitter acts on ligand-gated ion channels at the postsynaptic membrane to generate a postsynaptic potential (PSP). Center: electrical transmission occurs via intercellular channels that provide a pathway of low resistance for the spread of currents between cells which are known as “gap junctions.” The currents underlying a presynaptic action potential generate a coupling potential in the postsynaptic cell (coupling). Because most gap junctions conduct bidirectionally, the coupling potential is simultaneously transmitted to the presynaptic terminal. Right: electrical transmission can also occur as a result of the electric fields generated by neuronal activity. In this example, the electric field of an action potential that propagates and invades passively the presynaptic terminal generates an electric field that causes hyperpolarization at the postsynaptic cell. Modified from Pereda (2015), with permission.

The question of whether transmission between neurons is mediated electrically or chemically (Figure 1A) was posed formally in the 1870s, when the prevailing view of the nervous system was that it was a syncytium of connected nodes within a reticular structure. As stated by Eccles (Eccles, 1982), “It was an obvious conjecture that transmission between two electrically generating and responsive structures could be electrical,” but there already were experimental data suggesting chemical transmission at the neuromuscular synapse. The distinction between the two modes was clarified in the ensuing decades, with the advent of the neuron doctrine, according to which neurons are independent biological units (reviewed in Eccles, 1961, 1982). Briefly, the preponderance of data obtained at peripheral nervous system junctions was pharmacological and supported the concept of chemical transmission, such as the action of acetylcholine at the heart. However, in the case of the CNS, there wasn’t pharmacological data mimicking synaptic action, and the neuronal responses to applied chemical agents had longer delays than those of the responses evoked by nerve stimulation, leaving room to argue for electrical transmission.

Any model of electrical transmission must address a number of defining issues, including: (i) a mechanism for generating a postsynaptic signal strong enough to alter nerve cell excitability; (ii) a minimal synaptic delay, given the speed with which electricity travels in a conducting medium; and (iii) explaining how the same presynaptic signal, that is, an action potential, can produce excitation at some sites and inhibition at others. These three points are discussed separately below.

Fatt (1954) reviewed the two general mechanisms that could underlie electrical transmission. The first is a direct connection between the cytoplasms of the two coupled neurons via a low impedance path, with the degree of coupling being determined by the relative sizes of the coupling and “post-junctional” conductances. Although he considered this mode of transmission unlikely, coupling between nerve cells via gap junctions is now well-established, and these synapses can be uni- or bi-directional, depending on the voltage-dependent properties of the channel connexins (see below).

The second is “ephaptic” transmission or coupling via current flow through the extracellular space. This model dates back to experiments by Arvanitaki et al. (1964), who established artificial points of contact between two axons and showed current flow from one element to the next by applying an unbiologically powerful stimulus, the “detonator potential.” While there are numerous examples where the electrical activity of populations of neurons is modulated or biased by local extracellular fields (reviewed by Weiss and Faber, 2010), evidence for field effects that have characteristics analogous to those of chemical synaptic transmission has only been demonstrated in a few model systems. Nevertheless, these effects can be quite powerful. The best known examples involve the Mauthner cell, an identified reticulospinal neuron that triggers an escape behavior in many teleosts, and cerebellar Purkinje cells. In the former, ephaptic inhibition mediated by a specific class of interneurons sets the startle response threshold, and in the latter, it controls Purkinje cell synchrony. According to the ephaptic model, current associated with a presynaptic action potential is “forced” across the postsynaptic membrane because there is a high extracellular impedance in the surrounding neuropil. Thus, ephaptic transmission meets the first requirement listed above, namely, sufficient strength to be physiologically relevant, due to a specialized extracellular structure which is postulated to contribute to a high extracellular resistance. These specializations are known as the axon cap of the Mauthner cell and the pericellular basket, or Pinceau, of Purkinje cells. In the case of speed, suffice it to note that in these well-studied systems there is no delay between the simultaneously recorded presynaptic action potential and the “postsynaptic” field effect. Finally, whether a field effect is excitatory or inhibitory depends upon the direction and magnitude of postsynaptic current flow at the excitable postsynaptic membrane region, as discussed below. Here, we focus on the type of field effect that is analogous to chemical transmission, with identified pre- and postsynaptic elements, and the modulatory effects mediated by synchronous activation of populations of neurons are reviewed elsewhere (Weiss and Faber, 2010).

**Figure 2:**
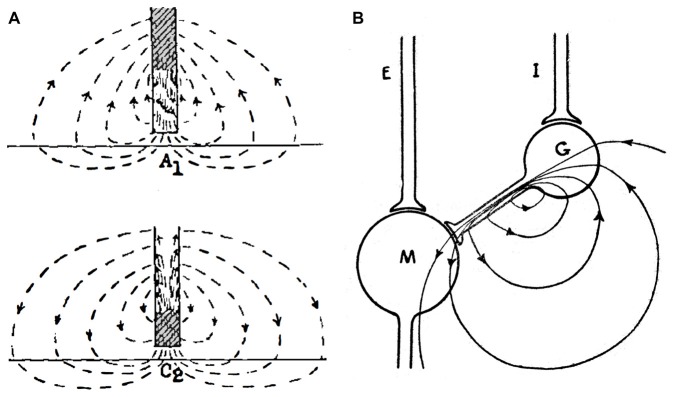
Proposed mechanisms for electrical transmission. **(A)** The cartoon illustrates the hypothetical current flow generated by an action potential approaching a synaptic terminal (top) and at the synaptic terminal itself (bottom). The initial anodal effect (A1) is followed by a cathodal effect (C2) in the postsynaptic membrane directly facing the presynaptic terminal. **(B)** Early electrical theory of inhibition. Cartoon illustrates the current flow through the synaptic terminal of an interneuron (G) on a postsynaptic cell (M). To exert an inhibitory action, the interneuron should receive subthreshold stimulation by its afferent input (I). An excitatory input (E) into the postsynaptic cell is also represented. Reproduced from Eccles (1982), with permission.

Interestingly, Eccles, who was a major proponent of electrical transmission in the CNS until he provided, with Fatt (1954), the most compelling evidence for the chemical mode, proposed models for electrical excitation and inhibition in the 1940s (Figure 2) which are still relevant today (Eccles, 1946; Brooks and Eccles, 1947). The models for electrical excitation and inhibition are quite straightforward; current from an extracellular source, e.g., the presynaptic axon, depolarizes and hyperpolarizes different regions of the postsynaptic membrane, with the constraints that: (i) the sum of imposed current flowing in across the neuronal membrane equals the sum of the outward current; and (ii) the functional sign of a field effect depends upon the direction of current flow across excitable postsynaptic membrane. The model proposed for electrical excitation postulated that a monophasic presynaptic current entered inexcitable postsynaptic membrane apposed to the synaptic terminal and exited across adjacent excitable membrane, thereby depolarizing the latter (Figure 2A). For electrical inhibition, sign inversion was achieved by interjecting an inhibitory interneuron that is depolarized but not to threshold, with its current in turn hyperpolarizing the inexcitable region of the postsynaptic membrane (Figure 2B). Eccles recognized that the current would be excitatory elsewhere and suggested that the extensive neuronal dendritic tree served the function of dissipating the outward excitatory current across a large distributed area of membrane, thereby minimizing its effect on excitation. These models, with the addition of distinguishing effects of membrane capacitance and implications of presynaptic spike waveform, account for most features of ephaptic transmission.

Finally a set of elegant experiments by Katz, Fatt, Miledi and colleagues showed that chemical transmission is mediated by a Ca^++^-dependent electrically regulated form of release of neurotransmitter packets (Katz, 1969), which in turn are capable of generating an electrical signal in the postsynaptic cell by acting specifically on ligand-gated ion channels known as “receptors” (Figure 1B, left). It is now recognized that both modes of communication, electrical and chemical, are operative (Figure 1B).

## Synaptic Transmission Mediated by Pathways of Low Resistance: Gap Junctions

As discussed above, Paul Fatt suggested that electrical currents generated in one neuron could directly spread to an adjacent postsynaptic cell via a pathway of low resistance. This idea led to the demonstration that, as postulated, presynaptic electrical currents can at some contacts propagate to the postsynaptic cell “electrotonically.” Moreover, not only action potentials (as are most often required for chemical transmission) but also subthreshold signals were conducted to the postsynaptic cell. In other words, changes in the membrane potential in one cell were capable of spreading to a second cell, generating potentials of similar time course but smaller amplitude, as if the two cells were “electrically coupled.” Electrotonic transmission was observed in both invertebrate (Watanabe, 1958; Furshpan and Potter, 1959) and vertebrate (Bennett et al., 1959; Furshpan, 1964) nervous systems.

**Figure 3:**
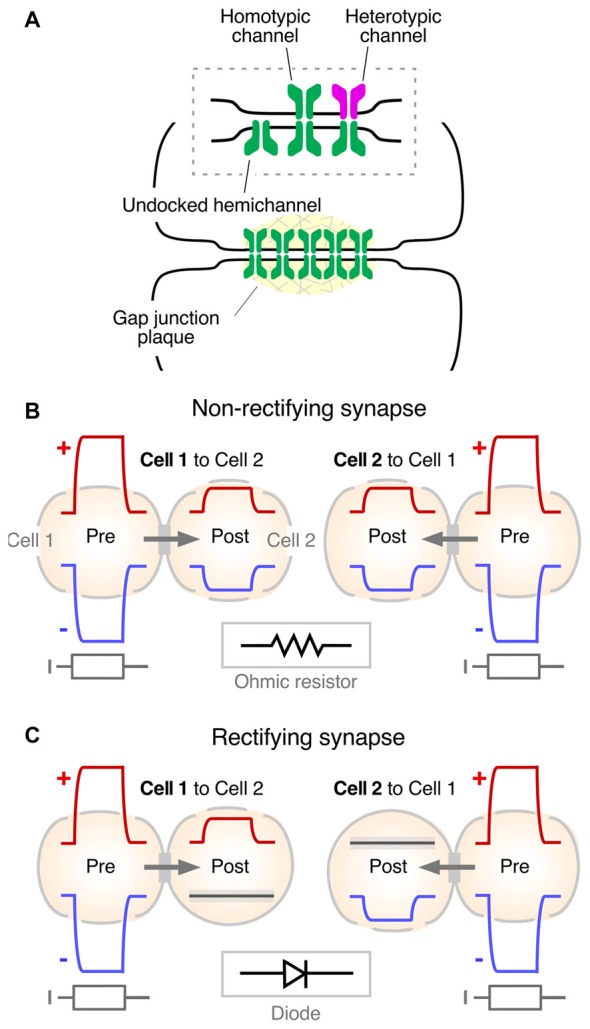
Synaptic communication mediated by gap junctions. **(A)** Gap junctions (Gap junction plaque) are groups of intercellular channels that provide a pathway of low resistance for the spread of electrical currents between two communicated cells. Inset: the intercellular channel is formed by the docking of two single channels (undocked hemichannel). The intercellular channel could be “homotypic,” at which both hemichannels are formed by the same gap junction channel-forming protein, or “heterotypic,” in which hemichannels are formed by different gap junction channel-forming proteins. Modified from Miller and Pereda (2017), with permission. **(B)** Non-rectifying electrical synapse. Both depolarizations (+, red traces) and hyperpolarizations (−, blue traces) evoked by intracellular current injection (I, gray traces) propagate to the postsynaptic cell in both directions (Cell 1 to Cell 2 and Cell 2 to Cell 1). Inset: the electrical behavior of most electrical synapses in physiological contexts correspond to that of an ohmic resistor (resistor symbol). **(C)** Rectifying synapse. Depolarizations, but not hyperpolarizations, propagate from Cell 1 to Cell 2. Conversely, hyperpolarizations, but not depolarizations, propagate from Cell 2 to Cell 1. Inset: in electrical terms, strongly rectifying electrical synapses behave as electric diodes (diode symbol).

Seminal experiments in fish (Robertson et al., 1963; Robertson, 1963; Furshpan, 1964; Pappas and Bennett, 1966; reviewed in Pereda and Bennett, 2017) led to the identification of the intercellular structure that serves as a pathway of low resistance for the spread of currents between neurons: the “gap junction.” Convergent evidence for the role of these structures in mediating electrical coupling was obtained in the heart (reviewed in Delmar et al., 2004). Gap junctions are groupings of tightly clustered intercellular channels (Figure 3A) that allow diffusion of intracellular ions carrying electrical currents (Goodenough and Paul, 2009). The intercellular channel is formed by the docking of two apposed individual channels, named “hemichannels” or “connexons,” one contributed by each of the coupled cells (Figure 3A). Hemichannels are hexamers made of connexins, a family of 21 genes in humans. Gap junctions are not exclusive to neurons, and they are present in virtually every tissue of an organism, acting as aqueous pores for metabolic support and chemical signaling (Goodenough and Paul, 2009). Only a minority of the connexins (Cxs) are expressed in neurons: Cx36, Cx45, Cx57, Cx30.2 and Cx50 (Söhl et al., 2005; O’Brien, 2014; Miller and Pereda, 2017; Nagy et al., 2018). Amongst them, Cx36 (Condorelli et al., 1998) is considered the main gap junction protein supporting electrical transmission in vertebrates. Except for microglia (Dobrenis et al., 2005) and other cells of ectodermic origin such as pancreatic beta cells (Moreno et al., 2005) and chromaffin cells (Martin et al., 2001), its expression is restricted to neurons (Rash et al., 2000). Combined, its widespread distribution and neuronal preference make Cx36 and its vertebrate orthologs the main channel-forming protein of neuronal gap junctions. Interestingly, a similar clustered organization of intercellular channels was found at invertebrate gap junctions, where the channels are formed by a different protein named “innexin,” a family of about 20 genes in *C. elegans* and 8 genes in the fly (Phelan et al., 1998; Phelan, 2005). Innexins form either hexameric or octameric hemichannels (Oshima et al., 2016; Skerrett and Williams, 2017). Remarkably, despite their unrelated sequences, connexins and innexins share a similar membrane topology and converge into similar structures with largely overlapping functions (Pereda and Macagno, 2017; Skerrett and Williams, 2017). There is a family of three genes found in vertebrates that share sequence similarities with innexins, the so-called “pannexins” (Panchin et al., 2000). Pannexins were found to be expressed in neurons (Bruzzone et al., 2003; Thompson et al., 2008), although there is no evidence so far indicating they form gap junctions *in vivo* and are capable of supporting electrical communication between neurons. Rather, they are thought to contribute functionally, operating as hemichannels (Dahl and Locovei, 2006; MacVicar and Thompson, 2010).

From the functional point of view, gap junction channels most commonly operate electrically as ohmic resistors, providing bidirectional communication for electrical signals between two or more cells (Figure 3B). Currents underlying action potentials in a presynaptic cell can directly flow via the gap junction to the postsynaptic cell, generating “electrical synaptic potentials” or “coupling potentials,” which also are known as “spikelets” (Figure 1B, middle). Not only currents underlying action potentials but also those responsible for subthreshold signals such as synaptic potentials of either depolarizing or hyperpolarizing nature can spread to the postsynaptic cell to generate a coupling potential (Figure 3B). The strength or weight of the postsynaptic cell’s response and the passive properties of the coupled cells are largely interdependent (Bennett, 1966; Getting, 1974). Accordingly, the amplitude of the coupling potential is determined not only by the conductance of the gap junction channels but also by the input resistance of the postsynaptic cell (see Bennett, 1966). In addition, the passive properties of the postsynaptic cell impose limitations to the transmission of presynaptic signals, depending on their duration. Short lasting signals such as action potentials are more attenuated than longer lasting signals such as synaptic potentials or afterhyperpolarizations due to the filtering properties of the postsynaptic membrane which are reflected by the membrane “time constant” of the cell (a parameter determined by the product of the cell’s resistance and capacitance that expresses how rapidly the resting membrane potential of the cell can be modified by a given current). As a result, the “coupling coefficient,” a measure of the synaptic strength, defined as the ratio between the amplitude of the postsynaptic coupling potential and that of the presynaptic signal, can be dramatically different for signals with different time courses.

Rather than simple conduits the gap junction channels themselves contribute to electrical communication. The molecular composition and properties of the gap junction intercellular channel have been shown to endow electrical transmission with voltage-dependent properties. Hemichannels that contribute to form the intercellular channel can be made of the same or different connexin or innexin proteins. Intercellular channels formed by hemichannels made of the same protein are called “homotypic,” whereas channels formed by hemichannels made of different proteins are called “heterotypic” (Figure 3A, inset). Molecular differences between the involved hemichannels are commonly associated with rectification of electrical transmission (Barrio et al., 1991; Verselis et al., 1994) and, providing support for such prediction, this association has been observed for both connexin (Rash et al., 2013) and innexin-based electrical synapses (Phelan et al., 2008). Rectification refers to the ability of electrical currents to preferentially flow in one direction, in other words, they behave as electrical diodes. However, this property critically depends on the polarity of the signal. As observed in the crayfish giant fiber synapses (Furshpan and Potter, 1959; Giaume et al., 1987), depolarizations can travel from the presynaptic to the postsynaptic side but not in the opposite directions, and hyperpolarizations can travel from the postsynaptic to the presynaptic side but not the other direction (Figure 3C). The polarized features of electrical transmission suggest the existence of a voltage-sensitive mechanism underlying this property. Several mechanisms were proposed to contribute to steep electrical rectification of gap junction channels, such as that observed in crayfish. Electrical rectification can be a consequence of the separation of fixed positive and negative charges at opposite ends of heterotypic gap junction channels, configuring a “p-n junction,” which results from asymmetries in the molecular composition of the hemichannels that form the intercellular channel (Oh et al., 1999). Alternatively, electrical rectification could result from the presence of charged cytosolic factors which alter channel conductance, such as Mg^++^ (Palacios-Prado et al., 2013, 2014) and spermine (Musa et al., 2004), which were to shown to interact with the gap junction channel. Combinations of these or more factors are likely to contribute to this striking voltage-dependent feature of some electrical synapses (reviewed in Palacios-Prado et al., 2014). Finally, the conductance of neuronal gap junctions was shown to be target of numerous regulatory mechanisms that endow electrical synapses with plastic properties equivalent to those observed at chemical synapses (reviewed in Pereda et al., 2013; O’Brien, 2014, 2017; Pereda, 2014).

## Synaptic Transmission Mediated by Electric Fields

**Figure 4:**
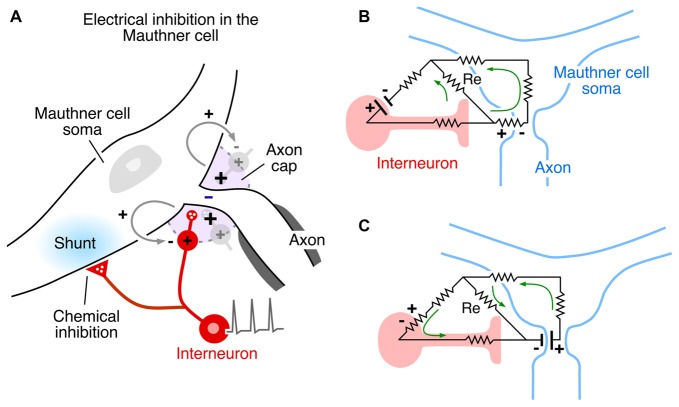
Inhibitory synaptic action in the Mauthner cell network mediated by electric fields. **(A)** Mixed electrical and chemical inhibition of the Mauthner cell mediated by action potentials in axonal endings of identified inhibitory interneurons (red). Some axon branches converge on the Mauthner cell’s Axon cap (violet) around its initial segment, and their action currents generate a hyperpolarizing extracellular positivity in the cap. The interneuron’s axons within and outside the cap are glycinergic and mediate chemical inhibition of the Mauthner cell, manifest as a postsynaptic shunt (blue regions). Modified from Pereda and Faber (2011), with permission. **(B,C)** Resistive circuit models demonstrating current flow associated with electrical inhibition of the Mauthner cell **(B)**, and of the inhibitory interneuron. **(C)** When the interneuron is activated, its action current is channeled through the axon and in across the Mauthner axon’s initial segment, generating an extracellular positivity in the axon cap, thereby hyperpolarizing the axon. When the Mauthner axon’s initial segment is activated, its action current is directed inward across the interneuron’s excitable membrane and returns to the source through the inexcitable terminal axon. Panels **(B,C)** modified from Faber and Korn (1989), with permission.

Theoretically, simple electrical circuits with biologically realistic constraints on the passive and active voltage-dependent properties of neurons, their spatial orientation and the conductivity of the extracellular space could be used to predict whether a single neuron or a group of synchronously active cells generate enough extracellular current to affect the excitability of neighboring cells. That small capacitive and ohmic currents do flow from one cell to the next is not in doubt (Figure 4). The question is whether the small fraction of the source current that will be channeled transcellularly is large enough to have functional significance? Weiss and Faber (2010) addressed that question by comparing the strengths of local field potentials (LFPs) associated with endogenous electrical activity of normal and epileptogenic hippocampal pyramidal neurons with the strengths of applied fields shown to modify the timing of spike activity, *in vitro*. The effective applied fields were weaker, consistent with the notion that fields effect rhythmogenesis and neuronal synchrony. This function is most likely exerted in homogeneous CNS structures where a population of neurons have similar morphologies and orientations, such that their currents sum, as for hippocampal and cortical pyramidal cells. Indeed, modeling combined with electrophysiological experiments suggest these modulations of ongoing activity may have functional significance (see, for example, Fröhlich and McCormick, 2010; Anastassiou et al., 2011; Berzhanskaya et al., 2013; Han et al., 2018).

But, can this mechanism also underlie synaptic communication? Contrasting Eccles’ models of ephaptic excitation and inhibition suggests that the former is relatively straightforward and is primarily a function of the parallel or radial alignment of a population of neighboring neurons and the conductivity of the extracellular space, i.e., of the relative impedance and the orientation of the transcellular and extracellular current pathways. Yet, there are no compelling examples of ephaptic excitation mediating a distinct synaptic function with identified pre- and postsynaptic elements. Indeed, it is surprising that the prominent examples of electrical interactions between neurons consistent with a synaptic function are inhibitory. They include the bidirectional inhibition between the teleost Mauthner cell and a class of inhibitory interneurons (Faber and Korn, 1973; Korn and Faber, 1976; Korn et al., 1978), and the connection between cerebellar Basket cells and Purkinje cells (Korn and Axelrad, 1980; Blot and Barbour, 2014). Furthermore, these model systems share structural specializations and physiological properties, lending support to the hypothesis that these examples represent a form of electrical synaptic action.

The Mauthner cell is a large identifiable midbrain neuron found in many teleosts, and it has a number of morphological specializations that make it an unique model system. Furukawa and Furshpan (1963) discovered the first example of electrical inhibition when comparing the intra- and extracellular potentials evoked in the axon cap by antidromic stimulation of this neuron’s axon—as noted, the axon cap is a dense neuropil surrounding the initial segment of the Mauthner cell axon. First, the antidromic action potential in the extracellular space (Ve) is very large and negative, as much as −40 mV, and the corresponding spike height recorded intra-axonally (Vi) at the site of spike initiation is smaller, ~+50 mV, so that the full transmembrane spike height, calculated as the difference between the intra- and extracellular responses, i.e., Vi − Ve, ~+90 mv (Furshpan and Furukawa, 1962). This observation of such a large extracellular potential associated with one neuron’s action potential suggested a high resistance barrier to extracellular current, and it has been proposed that this property is a consequence of the structure of the axon cap: swelling of interneuron axons at the edge of the cap, and close proximity to a densely packed ring of glia at the same boundary, known as the “canestro” or “basket” of Beccari (1907). These morphological features represent cellular specializations that support electrical communication and, therefore, may be analogous to structural specializations found at chemical synapses. Furthermore, the antidromic spike was succeeded by an extracellular positivity, which they named the Extrinsic Hyperpolarizing Potential (EHP) since it was larger than its intracellular representation, and, thus the same calculation showed that (Vi - Ve) < 0 and that the EHP is inhibitory. It was shown subsequently that the EHP was generated by impulses in a class of inhibitory interneurons that mediate feedback and feedforward inhibition of the Mauthner cell and that the evoked inhibition has two components, with a classical glycinergic inhibition of the Mauthner cell following the electrical component by ~0.5 ms (Figure 4A; Korn and Faber, 1976). In the case of the feedforward circuit, the short latency allows electrical inhibition to occur synchronously with excitation, thereby limiting the duration of the decision-making window in processing information by the Mauthner cell. Thus, these connections mediate mixed, electrical and chemical, synaptic actions (Figure 4A).

Additional specializations support the notion that electrical inhibition is physiological and functionally relevant. For example, the presynaptic spike in the inhibitory interneurons propagates passively within the cap, where the afferent axon loses its myelination. Consequently, the local field is monophasic, increasing its effectiveness. The EHP, which acts as an extracellular anode, that is, as an external current source, can be as large as 20 mV. Paired pre- and postsynaptic recordings show that the contribution of a single interneuron is about 0.4 mV per presynaptic spike, suggesting about 50 interneurons discharge synchronously following antidromic stimulation This is a powerful population effect that shuts down the Mauthner cell for 10’s of milliseconds. However, as noted above, these neurons are also excited in a feedforward circuit that relays auditory information to the Mauthner cell. In this case the EHP is graded as a function of stimulus strength, and it serves to set the threshold of a sound-evoked behavior, the escape response: when a sound-evoked EHP is canceled by an applied cathodal current in the axon cap, the underlying subthreshold EPSP is converted to suprathreshold, triggering Mauthner cell activation (Weiss et al., 2008).

Other factors which influence the operation of electrical inhibition include the orientation of the involved neurons and the distribution of excitable membrane relative to extracellular current sources and sinks. The Mauthner cell system is an ideal model for extracting mechanistic features, especially since there is reciprocal inhibition in the network, that is, the interneurons are inhibited by the Mauthner cell action currents (electric currents that originate from variations of potential during neural activity). Figures 4B,C contrasts the two examples. In both cases, the inhibitory current is channeled inward across excitable membrane, namely the Mauthner axon initial segment (Figure 4B) or the last node, or heminode, of the inhibitory interneuron’s axon (Figure 4C). Conversely, it exits the target through inexcitable membrane, that is, across soma-dendritic- or axon terminal membrane, respectively. Thus, if the distribution of excitable or inexcitable membrane were altered, the sign and magnitude of the ephaptic action would be altered accordingly. These considerations pertain to other networks as well, as discussed below.

**Figure 5:**
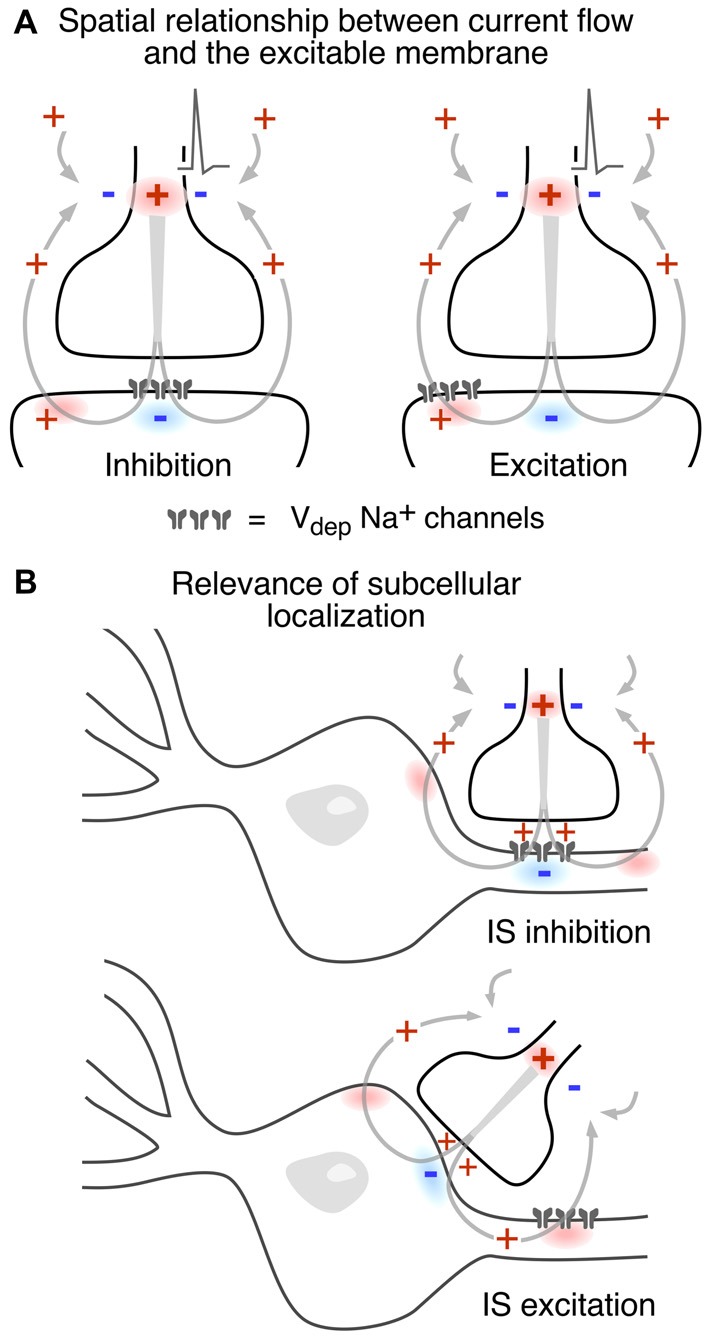
Presynaptic electric fields can exert both excitatory and inhibitory actions on a postsynaptic cell. **(A)** Whether an ephaptic current is excitatory or inhibitory depends on both the direction of current flow and the properties of postsynaptic membrane. The schematic model contact establishes the same currents in both examples, but excitable postsynaptic membrane, depicted as a cluster of voltage-dependent Na^+^ channels, is either restricted to the contact zone, in the case of electrical inhibition, or is displaced laterally, for electrical excitation. **(B)** The subcellular localization of presynaptic contacts also influences the polarity of an ephaptic synapse. Upper and lower schemes contrast axo-axonic and axo-somatic “electrical” synapses, respectively. The former is inhibitory because an inward hyperpolarizing current is imposed upon excitable postsynaptic membrane while the latter is instead excitatory because the current across the postsynaptic excitable membrane is outward.

Since the consequences of different spatial and functional arrangements are not necessarily intuitive, Figure 5 illustrates different combinations. In Figure 5A an inward postsynaptic current is inhibitory if the postsynaptic membrane is inexcitable at the site of contact, and it can be excitatory if the obligatory outward current exits through excitable membrane. The two examples in Figure 5B contrast the inhibitory and excitatory field effects generated at axo-axonic and axo-somatic contacts, respectively, when the excitable membrane is restricted to the axonal initial segment. It should be noted that these general examples do not factor in the dependence of the strength of the corresponding electrical interaction on the density and spatial distribution of current flow.

It is noteworthy that another system with well-studied ephaptic inhibition is the cerebellar pinceau where terminal axons of basket cells form a densely packed sheath around the Purkinje cell initial axon segment. This unusual axonic arrangement of inhibitory basket cells on Purkinje cells can be also considered, as in the Mauthner cell, a synaptic specialization supporting electrical transmission. Blot and Barbour (2014) showed that this ephaptic inhibition could, at very weak fields produced by a spike in a single basket cell, reduce the firing rate of an active Purkinje cell. They postulated that the coupling between cells was due to capacitive current flow, not to resistive current as suggested for the Mauthner cell. However, the time constants of the Mauthner cell and of the inhibitory interneurons are unusually brief, in the range of 100–200 microseconds, and 2 milliseconds, respectively, suggesting resistive coupling is a major portion of the electrical inhibition in that network. Regardless, the speed of coupling in both systems may be one function of electrical inhibitory synapses.

Evidence is slowly accumulating that ephaptic currents generated by single neurons can be detected, and they have been shown to influence neuronal firing patterns in diverse structures, including teleost midbrain (2005), mammalian cortex (Anastassiou et al., 2011), cerebellar cortex (Blot and Barbour, 2014) and snail CNS (Bravarenko et al., 2005). These findings suggest that physiologically relevant ephaptic interactions may be more ubiquitous than appreciated, a prospect supported by formal models of ordered structures, such as olfactory nerve (Bokil et al., 2001) and other olfactory structures (Van der Goes van Naters, 2013) and retina (Byzov and Shura-Bura, 1986; Vroman et al., 2013) but see (Kramer and Davenport, 2015), subject to structural and biophysical constraints, including the properties discussed here.

## Summary

The nervous system relies on electrical signaling to perform the fast computations that underlie animal behavior. Not surprisingly, intercellular communication between neurons can be mediated not only by the action of chemical transmitters, but also by electrical signaling. In turn, electrical communication occurs via two main mechanisms: one involves pathways of low resistance between neighboring neurons that are provided by intercellular channels (gap junctions), while the second, which is generally less appreciated, occurs as a consequence of the extracellular electrical fields generated by neurons during electrical signaling. Electrical signals generated by one cell can thus modify the excitability of its neighbors via one, or both, of these mechanisms. As with chemical transmission, each of the two modes of electrical transmission depends upon distinctive structural specializations, namely gap junctions in one case and a dense high resistance neuropil in the other.

Ephaptic interactions are generally perceived as only occurring in a diffuse manner, particularly in situations where the activity of a group of neurons influences the excitability of its neighbors. These interactions were proposed to play physiological (LFPs) and pathological roles (seizure maintenance). On the other hand, there are examples where presynaptic cellular specializations are found in close proximity to specific regions of the postsynaptic cell. This is the case for the axon terminals of inhibitory interneurons which impinge on the Mauthner cell, within the axon cap, and on cerebellar Purkinje cells, in the basket cell pinceau: in both cases these endings are located in close proximity to the initial segment of the “postsynaptic” cell. From our perspective, these two examples qualify as synapses, as presynaptic specializations enable localized actions on a very specific region of the postsynaptic cell. In addition to presynaptic anatomical specializations the interactions require an unusual high resistivity (or impedance) of the extracellular space. Moreover, these specializations were shown to be functionally and behaviorally relevant. More than one set of structural and physiological conditions are consistent with the electric field modality of synaptic communication. That is, the required anatomical and functional specializations do not follow a general pattern and seem specific for each case, making the identification of new examples by anatomical means particularly challenging. However, the Mauthner cell axon cap and the basket cell pinceau on the Purkinje cell unambiguously represent synaptic specializations and constitute the focus of this review article.

Despite the predominance of electrical signaling in the nervous system, synaptic communication ubiquitously occurs via the interposition of a chemical messenger, or neurotransmitter, between synaptically-connected cells. Chemical communication is likely an evolutionarily earlier communication strategy, as it occurs between unicellular organisms (Li and Nair, 2012). However, chemical communication between neurons is controlled by and capable of generating electrical signals: evoked transmitter release requires presynaptic depolarization and neurotransmitter receptors generate postsynaptic electrical signals (Sheng et al., 2012). Given such a close interrelationship between electrical signaling and chemical communication, the latter was also called “electrochemical transmission” (Llinás, 2001).

Despite their dependence on electricity, these three forms of communication co-exist because their individual properties differentially contribute to the processing of information within circuits. Chemical synapses, in addition to having receptors that gate ligand-bound ion channels capable of generating changes in the membrane potential of the cell following activation, have metabotropic receptors capable of activating a variety of biochemical cascades. Activation of biochemical cascades can occur following the binding of neurotransmitter to either ionotropic and metabotropic receptors and could lead to long-term modification of synaptic and/or cellular properties and induction of gene expression (Sheng et al., 2012). Thus, chemical synapses have the ability to transform a presynaptic signal into a variety of spatial and temporal patterns, an adaptive property that contributes to a great extent to the diversity of synaptic communication in the brain.

In contrast, the lack of a measurable synaptic delay implies that electrical transmission may be better adapted to ensure fast processing of signals through neural networks. While the two forms of electrical transmission have in common a high-speed of synaptic communication they however seem to serve different roles in communication, from the network point of view. There are, so far, fewer examples of electrical communication mediated by electric fields and as a result, less is known of the underlying mechanism. It is, however, known that its actions are localized on critical subcellular locations, such as the neuron’s initial segment. This feature allows synapses mediated by electric fields to convey exquisite timing information to decision-making cells within a circuit. Because of its speed, electrical transmission mediated by fields was shown to be critical in the processing of auditory information by the circuits that control the excitability of the teleost Mauthner cell (Weiss et al., 2008), and this mechanism is likely to play similar roles in controlling the activation of Purkinje neurons by cerebellar circuits (Blot and Barbour, 2014). In contrast, electrical synapses mediated by gap junctions are more widely distributed within neural networks and, a result of their bidirectionality, promote coordinated network activity by allowing computation of subthreshold variations of membrane potential between electrically-coupled cells. While promoting electrical synchronization is the signature property of gap junction mediated electrical synapses, their functional roles also include desynchronization, enhancement of signal to noise ratio, and coincidence detection, amongst others (reviewed in Connors, 2017).

Finally, the functional value of each form of transmission has its unique valence, which cannot be accomplished by the other. This functional categorization, is emphasized by the existence of mixed transmission at synaptic contacts at which chemical and electrical transmission, mediated by either gap junctions (Furshpan, 1964) or electric fields (Korn and Faber, 1976), act in concert to secure communication with a postsynaptic cell.

## Author Contributions

Both authors wrote the article.

## Conflict of Interest Statement

The authors declare that the research was conducted in the absence of any commercial or financial relationships that could be construed as a potential conflict of interest.
